# Systemic Lupus Erythematosus and Acute Transverse Myelitis: An Unusual Association—A Case Report

**DOI:** 10.1155/carm/9243188

**Published:** 2025-12-30

**Authors:** Baowêndsôm Armelle Djamilatou Sawadogo, Yannick Laurent Tchenadoyo Bayala, Ahmed Devis Wendyam Zougmoré, Marcellin Bonkoungou, Fulgence Kaboré, Wendlassida Joëlle Stéphanie Zabsonré/Tiendrébéogo, Dieu-Donné Ouédraogo

**Affiliations:** ^1^ Rheumatology Department of Bogodogo University Hospital Center, Ouagadougou, Burkina Faso

**Keywords:** sub-Saharan Africa, systemic lupus erythematosus, transverse myelitis

## Abstract

Acute transverse myelitis is a rare neurological complication of systemic lupus erythematosus (SLE), particularly in sub‐Saharan African origin patients. We report a case of acute transverse myelitis in a sub‐Saharan African origin patient with SLE. This case involved a 37‐year‐old female patient with SLE and secondary Sjögren’s syndrome, presenting with progressively worsening paraplegia. Examination revealed paraplegia of the lower limbs and paraparesis of the upper limbs. Lumbar puncture yielded clear cerebrospinal fluid with hyperproteinorachia. Spinal magnetic resonance imaging showed transverse myelitis in lumbar level; the 24‐hour proteinuria was 4.2 g. The diagnosis of acute transverse myelitis and renal flare of SLE was made. Clinical improvement was achieved with methylprednisolone, cyclophosphamide, and physiotherapy. Acute transverse myelitis remains a rare and poorly understood complication of lupus, characterized by its severity and very poor prognosis.

## 1. Introduction

Myelitis is a rare neurological condition caused by inflammation of the spinal cord, leading to motor, sensory, and sphincter impairments [1]. Lupus myelitis remains a rare and unusual complication of systemic lupus erythematosus (SLE) [1]. Acute transverse myelitis is its most characteristic presentation. Transverse myelitis complicates the course of SLE in less than 5% of cases [2]. Typically, this complication is considered an acute and severe manifestation with a poor functional prognosis and occurs late in the course of SLE [3]. Moreover, due to the low incidence of acute transverse myelitis associated with SLE, there are few African cases described in the literature concerning this pathology. To our knowledge, in sub‐Saharan Africa, only three cases have been recorded; one case was reported in Côte d’Ivoire among 29 SLE patients with neuropsychiatric symptoms and two cases were reported in Cameroon among 108 SLE patients with neuropsychiatric symptoms over a period of 10 years [4, 5]. The apparent underreporting of cases may partly reflect an underrecognition of SLE diagnosis in clinical practice. Also, acute transverse myelitis in SLE appears rarely reported in populations of sub‐Saharan African origin, possibly related to genetic background, environmental exposures, or disparities in access to care [4, 5]. Furthermore, the limited access to specialized rheumatology care in many African settings likely contributes to this underdiagnosis. Through this study, we report a case of acute transverse myelitis in a sub‐Saharan African origin patient followed for SLE.

## 2. Case Presentation

A 37‐year‐old woman, ANA, anti‐DNA, and anti‐Sm positive female, without family history of SLE or autoimmune disease, was admitted for progressive onset paraplegia evolving over one week in an afebrile context; there was no history of trauma or general health deterioration. She had been regularly followed for 6 years for SLE, initially diagnosed on the basis of photosensitivity, malar rash, polyarthritis, positive antinuclear antibodies, and anti‐dsDNA antibodies, associated with secondary Sjögren’s syndrome. She was treated with hydroxychloroquine at a dose of 400 mg per day, and her disease remained stable with a SELENA‐SLEDAI score of 0. On admission, physical examination reported a Glasgow score of 15; her general condition was preserved. Vital signs were within the normal range, with a blood pressure of 120/75 mmHg, heart rate of 78 beats per minute, respiratory rate of 16 breaths per minute, oxygen saturation of 99% on room air, and temperature of 36.8°C. Neurological examination found spastic paraplegia with an overall muscle strength of 1/5 in both lower limbs and paraparesis in the upper limbs with an overall muscle strength of 4/5. Deep tendon reflexes were abolished in the lower limbs; sensitivity was normal; there were no sphincter disorders. Examination of the cranial nerve pairs was normal; there were no coordination disorders. Lumbar puncture returned clear, “rock‐water” cerebrospinal fluid (CSF) with normal pressure. Examination of other organs and systems was normal.

**Figure 2:**
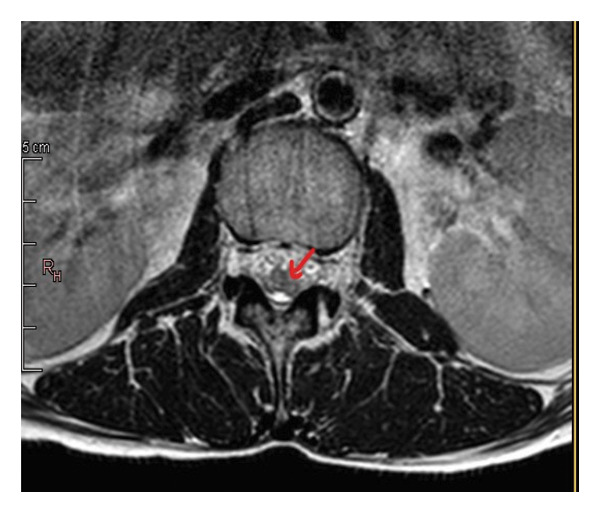
Spinal MRI, axial section with T2 weighting, showing a hypersignal occupying more than 50% of the spinal cord.

The hemogram showed inflammatory anemia with hemoglobin at 10.3 g/dL (normal: 12–16 g/dL), while leukocyte and platelet counts were within normal ranges. No lymphopenia was noted. C‐reactive protein was elevated at 74 mg/L (normal: < 5 mg/L), and the erythrocyte sedimentation rate was 36 mm at the 1st hour (normal: < 20 mm). Serum creatinine was 33 μmol/L (normal: 44–80 μmol/L). Muscle enzymes, liver function tests, and blood electrolyte levels were normal. The 24‐hour proteinuria was markedly elevated at 4.2 g/24 h (normal: < 0.3 g/24 h). Immunological markers such as serum IgG levels and complement fractions (C3, C4) were not available in our setting. Urinalysis did not show any bacteria. Chemical and cytobacteriological examination of the CSF revealed hyperproteinorachia at 1.2 g/L; glycorrhachia was normal and only one nucleated cell was found on cytology. CSF culture did not reveal any bacteria. Renal biopsy and antiphospholipid antibody testing were not performed. The electromyoneurography (ENMG) was normal. The SELENA‐SLEDAI score was 8, based on the presence of proteinuria > 3 g/24 h (4 points), anemia (hemoglobin < 11 g/dL) attributed to SLE (1 point), and a neurological manifestation (3 points). Spinal magnetic resonance imaging (MRI) showed hyperintensity on T2‐weighted and STIR images with contrast enhancement after gadolinium injection on T1‐weighted images at the lumbar level (Figure 1). These lesions extended over the entire lumbar spine, and in cross section, they affected more than 50% of the spinal cord (Figure 2).

Figure 1Spinal magnetic resonance imaging (MRI) of the lumbar spinal cord. (a) Sagittal T2‐weighted image showing extensive intramedullary hyperintensity involving more than 50% of the transverse diameter of the lumbar spinal cord. (b) Sagittal STIR image demonstrating intense and extensive intramedullary hyperintensity of the lumbar spinal cord. (c) Sagittal T1‐weighted image after gadolinium administration showing diffuse intramedullary contrast enhancement of the lumbar spinal cord.

**Figure:**
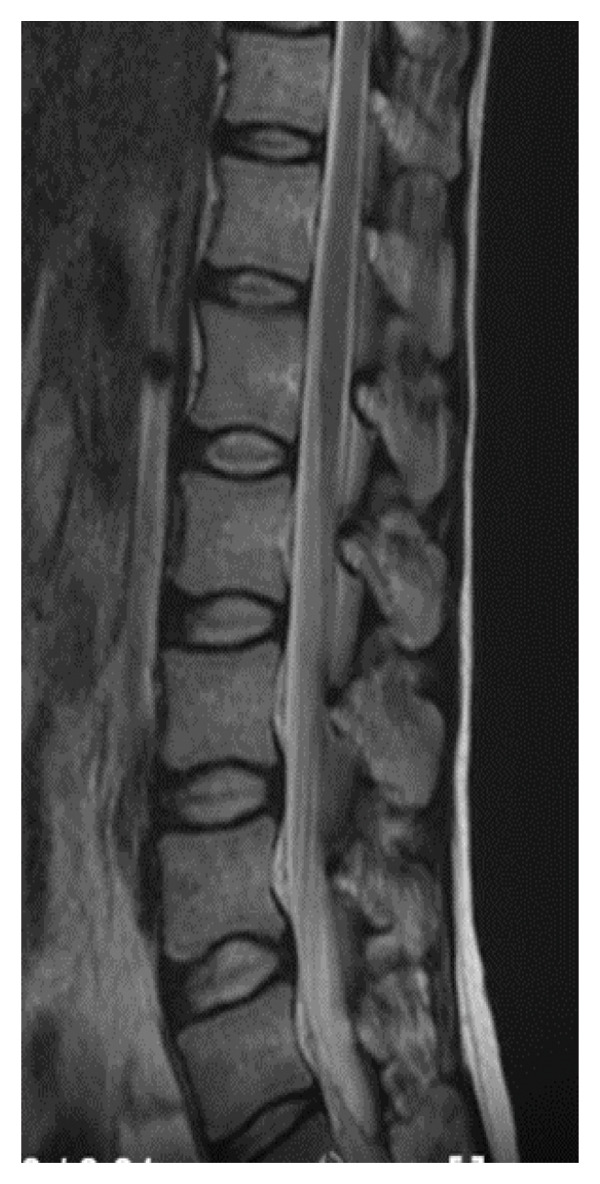
(a)

**Figure:**
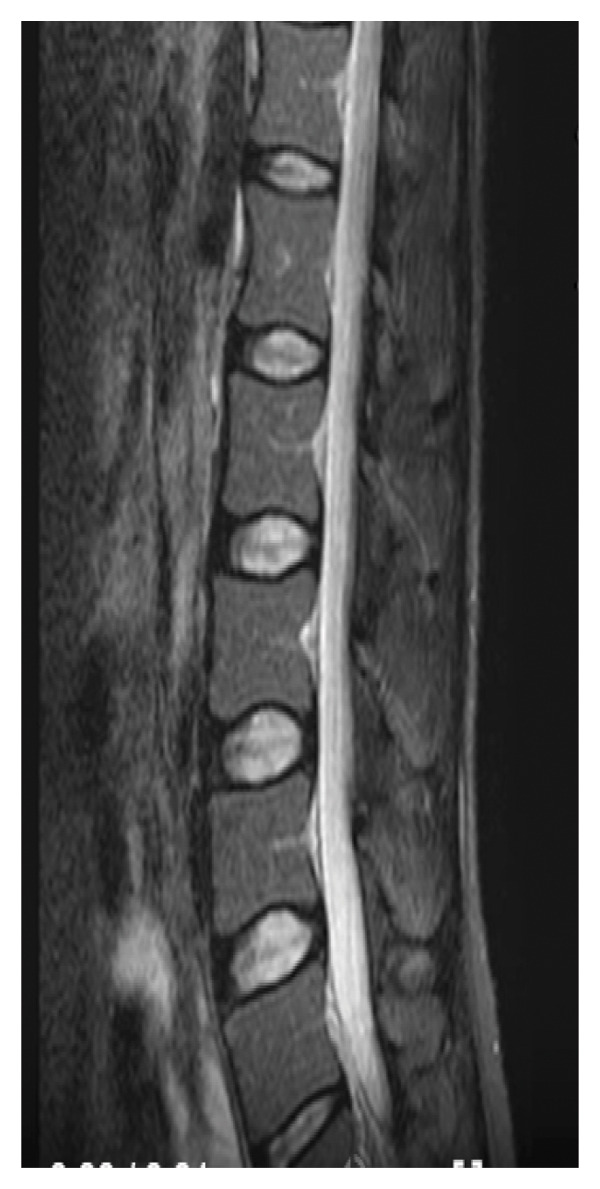
(b)

**Figure:**
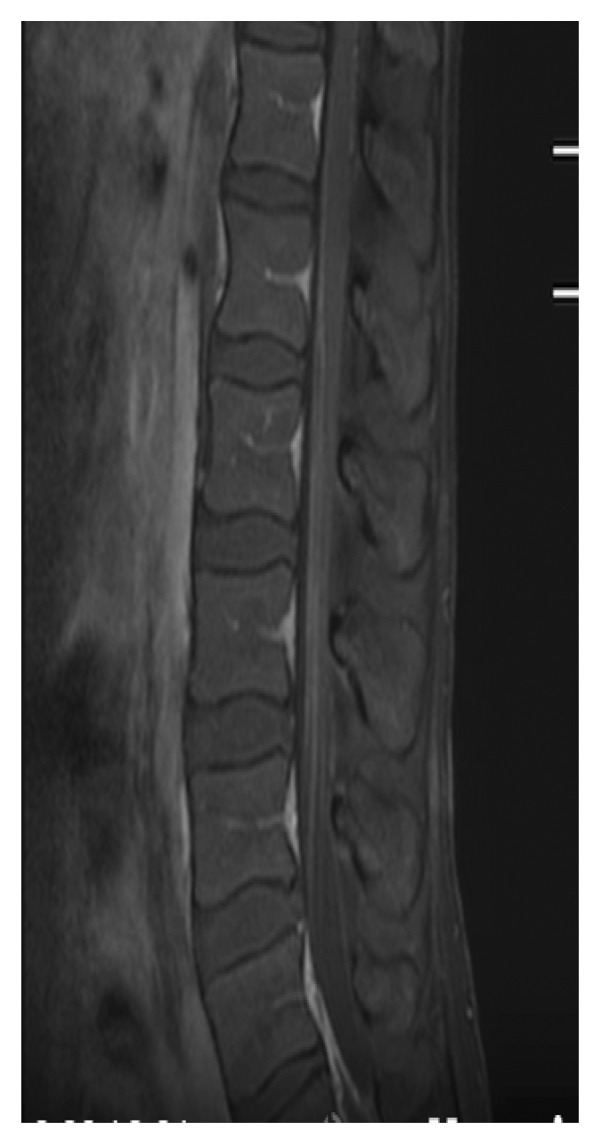
(c)

Given this clinical presentation and the MRI findings, the diagnosis of acute transverse myelitis was made in a lupus patient with nephropathy. The differential diagnoses considered in this patient included infectious myelitis, neuromyelitis optica spectrum disorder, multiple sclerosis, and other autoimmune or vasculitic causes of transverse myelitis.

The patient was treated with methylprednisolone at 240 mg/24 h for 3 days, followed by oral administration of prednisolone at a dose of 1 mg/kg/day, and cyclophosphamide IV according to the NIH protocol. Following cyclophosphamide, treatment was maintained with mycophenolate mofetil at a dose of 1 g twice daily and hydroxychloroquine at a dose of 200 mg twice daily.

By the 8th day, the motor deficit had improved with overall muscle strength at 3/5 in the lower limbs. At 1 month, there was a total recovery of motor function in all four limbs. Renal evolution was marked by normalization of 24‐hour proteinuria by the 6th cycle of cyclophosphamide. The SELENA‐SLEDAI score at the end of the treatment was 1.

A progressive corticosteroid tapering of 10% every 10 days was conducted over 6 months. Physiotherapy sessions were initiated during hospitalization and continued after discharge for 3 months, three times a week.

A repeat spinal MRI could not be performed due to financial constraints expressed by the patient, which limited the follow‐up imaging assessment. From the patient’s perspective, this acute neurological episode was a frightening and disabling experience, but she expressed great relief at her rapid neurological recovery and was grateful for the medical care received despite the limited resources available.

## 3. Discussion

Acute transverse myelitis represents, alongside demyelinating syndrome, the least prevalent and most severe neurological presentations among the twelve classic clinical manifestations of neurolupus recently defined by the American College of Rheumatology (ACR) [3]. Lupus‐induced acute transverse myelitis remains a rare and unusual complication of lupus, especially in sub‐Saharan African origin patient individuals. Indeed, Yacouba et al. in 2022 found in a 10‐year retrospective study in Cameroon 2 cases of acute transverse myelitis out of 108 lupus patients [4]. Additionally, Gbane et al. in 2019 in Côte d’Ivoire found only one case of acute transverse myelitis among the neurological manifestations of lupus patients in their series [5]. The etiological research is based on three contexts: infectious, autoimmune, and paraneoplastic [6]. Given the preexisting autoimmune context in our patient and the absence of evidence for an infectious or paraneoplastic cause, we attributed the acute myelitis to SLE. It is typically seen during the course of SLE, but it can also be the initial manifestation of the disease, as described by Zenone et al. [7].

A consensual clinical definition was proposed by Scott in 2007 and adopted by the French neurology group NOMADMUS in 2013 [8–10]. According to this definition, transverse myelitis clinically corresponds to a complete spinal cord syndrome combining a bilateral, more or less symmetrical, sensory‐motor deficit and sphincter disturbances [10]. However, the most commonly used definition is radiological with spinal MRI, which defines acute transverse myelitis as a lesion extending over at least 50% of the transverse surface of the spinal cord [9].

The etiopathogenesis of spinal cord dysfunction remains uncertain, although three types of hypotheses have been postulated in SLE‐related myelopathy [11]. First, the etiopathogenesis could be related to vasculitis; secondly, this myelitis could be explained by ischemic necrosis of the spinal cord; thirdly, there could be degeneration of the peripheral white matter [11]. This last hypothesis could be considered in our observation due to the spastic paraplegia indicating white matter involvement.

Spinal MRI plays a crucial role in the diagnosis of this pathology; it should explore the entire spinal cord. A sagittal section and a transverse section centered on the lesion should be performed. It generally shows, as in our observation, a heterogeneous T2 hypersignal at the spinal level [12]. Sometimes, it may only show an enlargement of the spinal cord or a normal appearance [12].

The functional prognosis of transverse myelitis is potentially severe with possible neurological sequelae [1]. It is not always unfavorable, as neurological recovery was possible in our observation. However, only 7% of patients in the series by Propper and Bucknall had a favorable functional prognosis [13]. In previously reported cases of SLE‐associated transverse myelitis, patients also exhibited additional clinical or serological evidence of active disease, supporting the notion that this complication often occurs in the context of global lupus activity [8–10].

There is no therapeutic consensus on transverse myelitis [12]. However, most authors currently emphasize the necessity of the rapid introduction of high‐dose corticosteroids and cyclophosphamide boluses, thus offering the best chances of neurological recovery [12]. Due to the rarity of lupus‐associated transverse myelitis, it is difficult to conduct controlled trials for treatment. Early and intensive neurological rehabilitation is an integral part of the management, even though no study has demonstrated its beneficial effect [14].

This study has several limitations that should be acknowledged. As a single case report, it inherently limits the generalizability of the findings to the broader population of patients with SLE. The absence of certain diagnostic investigations, such as complement levels, antiphospholipid antibody testing, and renal biopsy, restricted the comprehensiveness of the immunological and histopathological assessment. In addition, follow‐up spinal MRI imaging could not be performed due to financial constraints, preventing radiological confirmation of the patient’s neurological recovery. The lack of access to advanced diagnostic and monitoring tools, a common challenge in resource‐limited settings, may have introduced diagnostic uncertainty. Finally, the absence of long‐term follow‐up data limits the ability to evaluate sustained remission or potential relapse. Despite these limitations, this report contributes valuable clinical insight into an uncommon and severe neurological manifestation of SLE in a sub‐Saharan African context.

## 4. Conclusion

Acute transverse myelitis remains a rare and poorly understood complication of lupus, characterized by its severity and very poor prognosis. Early diagnosis and intensive, appropriate management are recommended to improve the outcome of this condition. Its treatment is not standardized, highlighting the need for large‐scale controlled trials to evaluate the efficacy of the described treatments. The primary take‐home message for clinicians is that maintaining a high index of suspicion for lupus myelitis, particularly in patients with known SLE presenting with acute neurological symptoms, is crucial to ensure timely recognition and management.

## Ethics Statement

The authors have nothing to report.

## Consent

Written informed consent was obtained from the patient to publish this case report and any accompanying data and images. A copy of the written consent is available for review by the Editor‐in‐Chief of this journal.

## Conflicts of Interest

The authors declare no conflicts of interest.

## Author Contributions

Writing–original draft preparation: Baowêndsôm Armelle Djamilatou Sawadogo; data curation: Yannick Laurent Tchenadoyo Bayala and Ahmed Devis Wendyam Zougmoré; writing, review, and editing: Marcellin Bonkoungou; and conceptualization and supervision: Fulgence Kaboré, Wendlassida Joëlle Stéphanie Zabsonré/Tiendrébéogo, and Dieu‐Donné Ouédraogo.

## Funding

No funding was received for this research.

## Data Availability

The data that support the findings of this study are available from the corresponding author upon reasonable request.
